# The Sleeping Habit

**Published:** 1893-03

**Authors:** 


### The Sleeping Habit.

Sleeping well should be as much a habit as bad sleeping. Early rising does much to favor sound sleep; and one secret of the perfect sleep of the country people is that they retire early and fatigued, and have perfect quiet around them. The conditions favoring sleep are perfect quiet, fatigue to more or less extent, darkness, a full meal, continuous monotony—as of a dull sermon or recitation, the quiet splash of the sea—freedom from pain, and warm extremities.

